# Increased blood glucose level following hysterectomy among reproductive women in India

**DOI:** 10.1186/s12905-020-01075-6

**Published:** 2020-09-23

**Authors:** Shiva S. Halli, Jang Bahadur Prasad, Rajeshwari A. Biradar

**Affiliations:** 1 Department of Community Health Sciences, Rady Faculty of Health Sciences, University of Manitoba, Winnipeg, MB R3E 0T6 Canada; 2 Department of Epidemiology and Biostatistics, KLE University, Belgaum, Karnataka 590010 India; 3 School of Development Studies, Tata Institute of Social Sciences, Mumbai, India

**Keywords:** Hysterectomy, Increased blood glucose, National Family Health Survey, India

## Abstract

**Background:**

In recent years, the hysterectomy, a surgical removal of the uterus, has received increased attention in health policy debates in India. The trigger for this was a series of media reports that highlighted an unusual surge in the number of women undergoing hysterectomies with a significant number of cases involving young and early menopausal women from low-income families. When menopause occurs as a result of hysterectomy, then the hormones such as estrogen and progesterone affect how the body cells respond to insulin. To date, we have not come across a national study following blood glucose levels among women who undergo a hysterectomy.

**Methods:**

The study used the Indian fourth round of National Family Health Survey data, which is a cross-sectional nationally representative sample of 699,686 women in the age group 15–49 years and conducted during 2015–16. Bivariate and multivariate logistic regressions were used to examine the effect of hysterectomy on blood glucose level of > 140 mg/dl among women of reproductive age groups.

**Results:**

The blood glucose level of > 140 mg/dl was much higher among women who had undergone a hysterectomy (12.2%) compared to non-hysterectomy women (5.7%). The pattern holds true among relevant background characteristics such as age, place of residence, education, caste, religion, wealth, marital status, body mass index (BMI), anaemia and consumption of tobacco. The adjusted odds after controlling for significant background factors, women who underwent hysterectomy experienced 15% higher odds of blood glucose level of > 140 mg/dl compared those who did not.

**Conclusions:**

The results indicated increased blood glucose level among women post hysterectomy. Hence, the government of India should consider developing evidence-based policies and programming to provide effective targeted interventions for the better reproductive health of women.

## Background

The surgical removal of woman’s uterus, hysterectomy, is becoming common in many countries. More than 600,000 hysterectomies are conducted in the United States each year [1]. In many countries hysterectomies are the second most common major operation in women of childbearing age [2]. In case of India, prevalence of hysterectomy is 3.2%, which vary by states/union territories, from 0.9 to 8.9% [3]. Some of the reasons for removal the woman’s uterus are fibroid tumours, endometriosis, and uterine prolapse. Approximately 44% of women have concomitant bilateral salpingo-oophorectomy (BSO) at the time of hysterectomy to prevent the subsequent development of ovarian cancer, treat medical conditions, or prevent the need for future adnexal surgery [1].

The surgical removal of woman’s uterus before end of reproductive period is significantly associated with death from all causes, and some have attributed it to a higher risk of cardiovascular disease [4, 5]. It has been postulated that women who undergo early hysterectomy may subsequently experience a higher risk of type 2 diabetes mellitus (hereafter referred to as diabetes) compared with women who do not undergo hysterectomy [6]. It is found that estrogens control of energy balance and glucose homeostasis show ovarian hormones regulate both insulin secretion and survival of pancreatic beta cells [7].

Epidemiological studies have shown associations between hysterectomy and diabetes risk elsewhere in the world. A study of 2597 postmenopausal in the United States reported that a hysterectomy was significantly associated with diabetes risk [8]. Also, earlier age at menopause and a shorter reproductive lifespan can lead to diabetes [9]. Another small, prospective study (only 33 hysterectomy women) found that women had a significant increase in fasting glycaemia in women with hysterectomies compared with women with natural menopause [10]. One rationale for this study was found in a British Broadcasting Corporation (BBC) documentary on hysterectomy highlighting that Doctors, especially in rural India, instead of using conservative techniques, went straight for surgeries which meant more money for them by exploiting the national health insurance scheme [11]. Reports from a few Indian states, including Rajasthan, Chhattisgarh, Bihar, and Andhra Pradesh, suggest that an unusually high number of women are having their uteruses removed, including many below the age of 40 [12]. For instance, the Oxfam report from one of the health camps stated that of 2606 women who were examined, 316 women, about 12% - had their uteruses removed unnecessarily [11]. However, this has adverse health effects both in the short term and long term [13]. Oxfam’s Health Policy Advisor reported the immediate health consequences such as incontinence, irritable bowel syndrome, back pain, depression, loss of sexual pleasure, thrombosis and vaginal prolapse [11, 13].

In the recent years, hysterectomy has received much attention in health policy debates in India. The reason for increased focus has been due to lots of media attention in highlighting significant increase in young women undergoing surgical removal of uterus in many states of India especially women from low-income families [11–13]. However, this may lead to an immediate decline in the production of sex hormones such as estrogen and progesterone, and in turn these affect how the body cells respond to insulin (7). A developing country like India, for effective targeting of health system resources and services especially with respect to non-communicable diseases, it is essential to understand whether hysterectomy will increase diabetes risk. This is particularly important as India is considered the diabetic capital of the world with more than 50 million Indians suffering from diabetes [14]. Recent study has shown that people living with diabetes in India increased from 26 million in 1990 to 65 million in 2016 [15]. Estimation showed that diabetes would be increasing from 73 million in 2017 to 134 million by 2045 in India for 20+ age [16]. About 25% of Indians above 18 years will add to future burden and risk of death due to diabetes/hypertension [17]. While wide variations by sex in the prevalence of diabetes mellitus have been documented in several articles [18–21], the causes of this heterogeneity need to be understood including the effect of hysterectomy on diabetes risk among reproductive women. To date no nationally representative study of increased blood glucose among women who undergo hysterectomy (hereafter referred to as hysterectomy women) and women who did not undergo hysterectomy (hereafter referred to as non-hysterectomy women) in India. Hence, the purpose of this study is an attempt towards addressing this research gap to provide much-needed evidence to inform policymakers.

## Methods and materials

### Sampling method

The NFHS is a large scale survey conducted by the International Institute for Population Sciences, and the detailed description of sampling design is provided in the published NFHS report [3]. The survey is based on a multi stage cluster sampling design using 2011 Census of India as a sampling framework to select primary sampling units to represent both rural and urban areas. In the second stage, households were selected from the selected primary sampling units in rural and urban clusters using a systematic random sampling design; and eligible women were selected from the selected households.

### Sample size and data

As indicated earlier the International Institute for Population Sciences conducted the survey and the study used the women’s file of National Family Health Survey round four (NFHS-4) 2015–16. This was a household survey covering 640 districts from 29 States and 6 Union Territories of India. Across the country, 28,586 Primary Sampling Units (PSUs) were selected, out of which 28,522 clusters fieldwork was completed. With a response rate of 98%, a total of 601,509 households were successfully interviewed by well trained interviewers in a private setting (e.g., house) to maintain confidentiality using Computer-assisted personal interviewing (CAPI) electronic device to answer the free installed structured questionnaire. A total of 723,875 eligible women age 15–49 were identified for an interview among interviewed households. With a 97% response rate, 699,686 women participated in the survey. However, we excluded pregnant women, and in the remaining sample of 667,258, blood glucose record was available only for 653, 002 women.

### Description of variables

The outcome variable is increased blood glucose level among hysterectomy and non-hysterectomy women in reproductive ages. The NFHS-4 collected random blood glucose using a finger-stick blood specimen using a freestyle optimum glucometer. Based on random glucose levels, NFHS-4 classified the women as ‘high’ for the level (141–160 mg/dl) and ‘very high’ for (> 160 mg/dl) [3]. However, some researchers defined the random cut-off of 2-h plasma glucose > 200 mg/dl (11.1 mmol/l) criterion is equivalent to the random capillary blood glucose cut-off point of 140 mg/dl (7.7 mmol/l) [22]. Similarly, others also defined a random glucose level of > 140 mg/dl as above normal and in this study, we too follow this definition as increased blood glucose level [3, 20, 21].

Based on the NFHS-4 report, the number of socioeconomic and demographic variables have been included to profile the sample [3]. More specifically, these variables are age (15–29, 30–39, and 40–49), place of residence (Rural and Urban), education level (No education, Primary, Secondary and Higher), type of caste (Scheduled Caste/Scheduled Tribe (SC/ST), other backward caste (OBC) and Others), religion (Hindu, Muslim and Others), wealth index (Poorest, Poor, Middle, Richer and Richest), and marital status (Never married, Currently Married, and Others), occupation (Not working and Working), drink alcohol (No and Yes), consumption of tobacco include both smoke’s tobacco (No and Yes) and use of smokeless tobacco (No and Yes) [3]. In addition to this, Body Mass Index (BMI) was categorized as too thin for their height (BMI below 18.5 kg/m^2^), normal (between 18.5–24.9 kg/m^2^), overweight (between 25 and 29 kg/m^2^) and obese (30 and above kg/m^2^)), and similarly anaemia was categorised as severe, moderate, mild and not anaemic) [3].

### Statistical analysis

IBM’s Statistical Package for the Social Sciences (SPSS) version-20.0 was used for the analyses. The bivariate analysis has been used to assess the association between the risk for diabetes among hysterectomy and non-hysterectomy women by background characteristics. Multivariate analysis has been used for assessing the effect of hysterectomy after controlling for background characteristics on the risk for diabetes among non-pregnant women. In case of categorical background characteristics, the choice of a reference category was guided by theoretical considerations as well the findings of bivariate analysis. For instance, in case of life style factors such as BMI, anaemia, smoking and drinking, we considered normal BMI, not anaemic, non-smokers and non-consumers of alcohol as reference categories. Similarly, for wealth quantile, place of residence, marital status and religion, the reference categories are poorest, urban, never married and Hindus respectively.

## Results

**Fig. 1:**
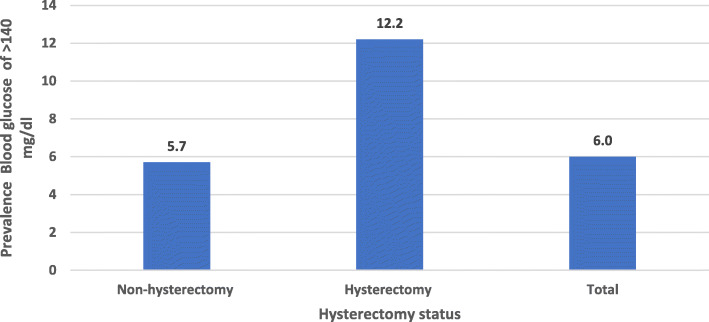
Prevalence of blood glucose of > 140 mg/dl by hysterectomy status among non-pregnant reproductive women in India, 2015–16

The prevalence of high blood glucose (> 140 mg/dl) among non-hysterectomy women was 5.7%, but it was 12.2% among hysterectomy women (Fig. 1). Table 1 reveals the high blood glucose level among reproductive women by important background characteristics including their hysterectomy status. Similarly, the prevalence was higher among older, urban, richer and working obese women compared to their counterparts (Table 1).

**Table 1:** Blood glucose of > 140 mg/dl by background characteristics and hysterectomy status among non-pregnant reproductive women in India, 2015–16

Background characteristics	Categories	Prevalence	*P*-value^a^	Total^b^
Age	15–29	2.8	.000	327,901
	30–44	7.7		253,995
	45–49	13.8		71,106
Type of place of residence	Urban	7.0	.000	190,253
	Rural	5.4		462,749
Religion	Hindu	5.9	.000	486,081
	Muslim	6.2		86,743
	Others	6.6		80,178
Caste	SC/ST	5.5	.000	234,978
	OBC	5.9		255,720
	Others	6.6		162,304
Marital Status	Never married	2.5	.000	166,794
	Currently Married	6.9		458,702
	Others	9.1		27,506
Wealth index	Poorest	4.4	.000	123,587
	Poorer	5.0		139,656
	Middle	5.6		138,057
	Richer	7.0		129,823
	Richest	7.4		121,879
Occupation	Not working	6.0	.005	78,430
	Working	6.4		33,925
Body Mass Index	Too thin for their height	3.3	.000	139,517
	Normal	4.7		382,836
	Overweight	10.6		91,481
	Obese	16.9		28,561
Drinks alcohol	No	6.0	.016	636,578
	Yes	5.9		16,424
Use of Smokes tobacco	No	5.9	.000	644,427
	Yes	7.8		8575
Use of Smokeless tobacco	No	5.9	.000	603,842
	Yes	7.8		49,160
Education level	No education	7.8	.000	185,183
	Primary	7.0		82,564
	Secondary	6.8		311,958
	Higher	5.4		73,297
Anaemia level	Severe	8.2	.000	6483
	Moderate	6.5		74,659
	Mild	5.6		255,556
	Not anaemic	6.1		316,233
Hysterectomy status	Non- hysterectomy	5.7	.000	635,215
	Hysterectomy	12.2		17,787
India		6.0		653,002

Note: ^a^ The Chi-square statistic is significant at .05 level; ^b^Unweighted cases

Table 2 reveals a comparative overview of the prevalence of risk for diabetes by background characteristics among hysterectomy and non-hysterectomy women in India. The table revealed the higher blood glucose level (> 140 mg/dl) among hysterectomy women compared to non-hysterectomy women for each category of background characteristics, sometimes nearly three times higher. For instance, among urban, educated, rich, widowed and obese hysterectomy women, the high blood glucose levels were 17.1, 18.6, 18.8, 14.5, 16.3% compared to non-hysterectomy women with levels of 6.8, 4.8, 7.0, 8.8, 25.7% respectively. As expected, age, wealth index, and BMI are all positively associated with the high blood glucose level and rural residence, SC/ST caste, Hindus and never-married women had lower diabetes risk compared to their counterparts for both groups of women (hysterectomy and non-hysterectomy) (Table 2).

**Table 2:** Blood glucose of > 140 mg/dl by background characteristics among non-hysterectomy and hysterectomy non-pregnant reproductive women in India, 2015–16

Background characteristics	Categories	Blood glucose among non-hysterectomy women			Blood glucose among hysterectomy women		
		Prevalence	P-value^a^	Total^b^	Prevalence	P-value^a^	Total^b^
Age	15–29	2.8	0.00	326,885	3.4	0.000	1016
	30–44	7.6		243,648	10.4		10,347
	45–49	13.4		64,682	16.7		6424
Type of place of residence	Urban	6.8	0.00	185,388	17.1	0.000	4865
	Rural	5.2		449,827	10.2		12,922
Religion	Hindu	5.6	0.00	471,588	11.8	0.000	14,493
	Muslim	6.0		84,880	14.8		1863
	Others	6.3		78,747	15.7		1431
Caste	SC/ST	5.3	0.00	229,982	11.0	0.002	4996
	OBC	5.6		247,444	12.3		8276
	Others	6.4		157,789	13.5		4515
Marital Status	Never married	2.5	0.00	166,723	10.6	0.001	71
	Currently Married	6.7		442,254	12.1		16,448
	Others	8.8		26,238	14.5		1268
Wealth index	Poorest	4.3	0.00	120,981	7.2	0.000	2606
	Poorer	4.9		136,112	9.4		3544
	Middle	5.4		134,006	9.9		4051
	Richer	6.7		125,822	14.4		4001
	Richest	7.0		118,294	18.8		3585
Occupation	Not working	5.8	0.00	76,477	14.1	0.107	1953
	Working	6.2		32,792	10.2		1133
Body Mass Index	Too thin for their height	3.3	0.00	137,266	5.0	0.000	2251
	Normal	4.6		373,679	8.7		9157
	Overweight	10.2		87,101	16.6		4380
	Obese	16.3		26,844	25.7		1717
Drinks alcohol	No	5.7	0.00	619,175	12.3	0.063	17,403
	Yes	5.8		16,040	9.2		384
Use of Smoke’s tobacco	No	5.7	0.00	626,910	12.3	0.111	17,517
	Yes	7.7		8305	8.6		270
Use of Smokeless tobacco	No	5.6	0.00	587,322	12.3	0.410	16,520
	Yes	7.7		47,893	11.8		1267
Education level	No education	6.8	0.00	176,245	10.6	0.000	8938
	Primary	6.5		79,592	12.9		2972
	Secondary	5.2		306,729	13.8		5229
	Higher	4.8		72,649	18.6		648
Anaemia level	Severe	8.1	0.000	6384	13.2	0.113	99
	Moderate	6.3		73,374	14.0		1285
	Mild	5.4		249,489	11.2		6067
	Not anaemic	5.8		305,898	12.6		10,335
India		5.7		635,215	12.2		17,787

Note: ^a^The Chi-square statistic is significant at .05 level; ^b^Unweighted cases

Because of possible confounding factors, the results of multivariate logistic regressions were presented in Table 3 to understand the effect of hysterectomy status on the blood glucose level of more than 140 mg/dl after controlling for the significant background variables. For comparative purpose, the table included both adjusted and unadjusted models. We suspect some interactions among some interrelated independent variables and hence, both the models, one with interaction terms and the other without, are presented for comparison. For instance, in case of unadjusted model, the odds ratios for all the background characteristics were significant, but in the adjusted model, the odds ratios were reduced for all most all the characteristics. In fact, for some variables such as education, place of residence, alcohol consumption and anaemia status the odds of high blood glucose were not significant at 0.05 level of significance. However, the odds ratios of important characteristics such as wealth status and BMI showed strong positive associations with the high blood glucose. To be specific, in case of wealth quantiles, rich women experienced 19% (95% CI 1.070–1.173, *p* = 0.0001) greater odds of high blood glucose (> 140 mg/dl) compared to poorest women. Similarly, in case of BMI, obese women experienced 146% (95% CI 2.034–2.979, p = 0.0001) greater odds of high blood glucose (> 140 mg/dl) compared to women with normal BMI. Moreover, for age and BMI, main effects as well as their interaction effects were significant. A conspicuous finding was that even after controlling for important background characteristics including age, BMI, anaemia and wealth status as well as the interaction effects among interrelated factors, those women who underwent hysterectomy experienced greater odds of 15% (95% CI 1.098–1.213, *p* = 0.0001) of high blood glucose (> 140 mg/dl) compared to those who did not.

**Table 3:** Adjusted and Unadjusted Odds Ratio for association of covariates with Blood Glucose > 140 mg/dl among non-pregnant reproductive women in India, 2015–16, India

Predictor Variables	Categories	$Adjusted Model			Unadjusted Model		
		Odds Ratio	p-value	95 C.I.	Odds Ratio	p-value	95 C.I.
Age		1.06	0.000	1.06–1.06	1.07	0.000	1.06–1.07
Education		1.00	0.232	0.99–1.01	0.97	0.000	0.97–0.97
Place of residence	Urban	Ref.			Ref.		
	Rural	0.99	0.604	0.95–1.03	1.31	0.000	1.29–1.34
Religion	Hindu	Ref.			Ref.		
	Muslim	1.07	0.000	1.04–1.11	1.07	0.000	1.04–1.11
	Others Religion	1.05	0.003	1.02–1.09	1.15	0.000	1.11–1.18
Marital status	Never Married	Ref.			Ref.		
	Currently Married	1.14	0.000	1.10–1.19	0.39	0.000	0.38–0.41
	Others	1.07	0.002	1.03–1.12	1.43	0.000	1.37–1.49
Wealth Index	Poorest	Ref.			Ref.		
	Poor	1.06	0.004	1.02–1.10	1.12		1.08–1.16
	Middle	1.10	0.000	1.06–1.15	1.27	0.000	1.23–1.32
	Rich	1.19	0.000	1.14–1.24	1.54	0.000	1.49–1.60
	Richest	1.12	0.000	1.07–1.17	1.68	0.000	1.63–1.74
BMI	Normal	Ref.			Ref.		
	Too thin for their height	1.07	0.313	0.94–1.21	0.76	0.000	0.73–0.78
	Overweight	1.45	0.000	1.27–1.65	2.42	0.000	2.36–2.48
	Obesity	2.46	0.000	2.03–2.98	4.18	0.000	4.04–4.33
Smokes tobacco	No	Ref.			Ref.		
	Yes	1.25	0.000	1.16–1.36	1.45	0.000	1.34–1.57
Smokeless tobacco	No	Ref.			Ref.		
	Yes	1.19	0.000	1.15–1.24	1.44	0.000	1.39–1.49
Alcohol Consumption	No	Ref.			Ref.		
	Yes	0.94	0.073	0.88–1.01	1.08	0.002	1.02–1.16
Anaemia status	Not Anaemic	Ref.			Ref.		
	Anaemic	1.03	0.058	0.99–1.06	0.95	0.000	0.93–0.97
Hysterectomy status	Non-hysterectomy	Ref.			Ref.		
	Hysterectomy	1.15	0.000	1.10–1.21	2.24	0.000	2.14–2.35
Interaction of BMI with Age	Too thin for their height by Age	0.99	0.000	0.99–0.99			
	Overweight by Age	1.01	0.000	1.01–1.01			
	Obese by Age	1.01	0.008	1.00–1.01			
Interaction of BMI with Rural	Too thin for their height by Rural	1.11	0.015	1.02–1.20			
	Overweight by Rural	0.93	0.006	0.88–0.98			
	Obese by Rural	0.89	0.002	0.83–0.96			
Interaction of BMI with Anaemia	Too thin for their height by anaemic	1.2	0.000	1.12–1.29			
	Overweight by anaemic	0.95	0.062	0.90–1.00			
	Obese by anaemic	0.99	0.727	0.92–1.06			

Note: $Model χ2 = 24.362, *p* < 0.01, C.I. – Confidence Interval, Ref. = Reference category

## Discussion

Hysterectomy has been a serious concern among policy makers in India. The reason for increased focus has been due to lots of media attention in highlighting significant increase in young women undergoing surgical removal of uterus especially from low-income families [11]. For effective targeting of health system resources and services, it was essential to understand how high blood glucose level (> 140 mg/dl) varies among hysterectomy and non-hysterectomy women based on a large scale nationally representative sample.

The study results showed that the overall high blood glucose level among non-pregnant reproductive women in India was 6.0%. However, the high blood glucose level was 12.2% among hysterectomy women and 5.7% among non-hysterectomy women. Surprisingly, the prevalence was consistently higher for each of the background characteristics considered in the study among the hysterectomy women compared to the non-hysterectomy women. The multivariate-adjusted odds ratios have also shown that the risk of diabetes due to hysterectomy increased significantly (15%) among women even after controlling for possible confounders such as body mass index, wealth index, age and place of residence.

The pathophysiology of the higher blood glucose among hysterectomy women remains elusive. Some of the reasons advocated in the literature regarding the higher blood glucose level among hysterectomy women are as follows: ovarian hormones, especially estrogens, play a pivotal role in the physiology of reproductive, skeletal and central nervous systems; while, ovarian hormones control body energy balance, hypothalamic nuclei in the liver, skeletal muscle, adipose tissue, and immune system cells, as they affect insulin sensitivity and exert anti-inflammatory effects [7, 8, 23, 24]. Other studies have shown that among hysterectomy women, there was higher progesterone which is associated with increased insulin resistance [25–27]. This is known as luteal phase insulin resistance leading to diabetes risk. Moreover, more resistance to insulin may likely cause food cravings for simple carbohydrates and may even cause women to lose motivation to exercise. This could further lead to poor glycaemic control. Over time, this cyclical poor control can increase the risk of diabetes.

Another argument in the literature is that hysterectomy might compromise ovarian blood flow from the ovarian ligament subsequently leading to early menopause. Diabetes risk is associated with the reduction of endogenous ovarian hormones in postmenopausal women. The confounding effects of early menopause and hormone changes likely to lead diabetes risk [24]. There are also other studies which indicate early menopausal women develop significantly more insulin resistance after hysterectomy in comparison to postmenopausal women which may lead to long-term effects of hysterectomy on diabetes disease in particular and women’s health in general.

There are some limitations of the study. For instance, the increased blood glucose due to hysterectomy might include persons with prediabetes, and therefore, the results should be interpreted with caution. Though it is difficult to establish clear causal links between hysterectomy and diabetes risk in cross-sectional data, it is worth the effort for the government to suggest surveillance to the general practitioners as well as obstetricians and gynaecologists following a hysterectomy so that evidence-based policies and programs can be developed.

## Conclusion

In the recent years in India, hysterectomy has been a topic of discussion not only among health researchers and also among policy makers. The main reason for this focus is due to heightened media attention in reporting young women undergoing hysterectomy especially from low-income families [11–13]. When menopause occurs as a result of hysterectomy, then the hormones estrogens and progesterone affect how the body cells respond to insulin. Women in India who undergo a hysterectomy at younger ages have a longer risk of exposure to high blood glucose leading to higher diabetes risk. The current study presents evidence on the increased blood glucose level after hysterectomy which may in turn lead to diabetes. Hence, the government of India should consider developing evidence-based policies and programming to provide effective targeted interventions for the better reproductive health of women.

## Data Availability

All the data that is used in this paper has been archived in the Demographic and Health Surveys (DHS) public repository, where the data is accessible using the link: https://www.dhsprogram.com/data/available-datasets.cfm.

## Abbreviations

NFHS: National Family Health Survey
BBC: British Broadcasting Corporation
IIPS: International Institute for Population Sciences
SC/ST: Scheduled Caste/Scheduled Tribe (SC/ST)
OBC: Other Backward Caste
BMI: Body Mass Index
BSO: Bilateral Salpingo-Oophorectomy
